# Hormonal response to lipid and carbohydrate meals during the acute postprandial period

**DOI:** 10.1186/1550-2783-8-19

**Published:** 2011-11-11

**Authors:** Rick J Alleman, Richard J Bloomer

**Affiliations:** 1 Cardiorespiratory/Metabolic Laboratory, Department of Health and Sport Sciences, University of Memphis, Memphis, TN, USA

## Abstract

**Background:**

Optimizing the hormonal environment during the postprandial period in favor of increased anabolism is of interest to many active individuals. Data are conflicting regarding the acute hormonal response to high fat and high carbohydrate feedings. Moreover, to our knowledge, no studies have compared the acute hormonal response to ingestion of lipid and carbohydrate meals of different size.

**Methods:**

We compared the hormonal response to lipid and carbohydrate meals of different caloric content during the acute postprandial period. Nine healthy men (22 ± 2 years) consumed in a random order, cross-over design one of four meals/beverages during the morning hours in a rested and fasted state: dextrose at 75 g (300 kcals), dextrose at 150 g (600 kcals), lipid at 33 g (300 kcals), lipid at 66 g (600 kcals). Blood samples were collected Pre meal, and at 0.5 hr, 1 hr, 2 hr, and 3 hr post meal. Samples were assayed for testosterone, cortisol, and insulin using ELISA techniques. Area under the curve (AUC) was calculated for each variable, and a 4 × 5 ANOVA was used to further analyze data.

**Results:**

A meal × time effect (p = 0.0003) was noted for insulin, with values highest for the dextrose meals at the 0.5 hr and 1 hr times, and relatively unaffected by the lipid meals. No interaction (p = 0.98) or meal (p = 0.39) effect was noted for testosterone, nor was an interaction (p = 0.99) or meal (p = 0.65) effect noted for cortisol. However, a time effect was noted for both testosterone (p = 0.04) and cortisol (p < 0.0001), with values decreasing during the postprandial period. An AUC effect was noted for insulin (p = 0.001), with values higher for the dextrose meals compared to the lipid meals (p < 0.05). No AUC effect was noted for testosterone (p = 0.85) or cortisol (p = 0.84).

**Conclusions:**

These data indicate that 1) little difference is noted in serum testosterone or cortisol during the acute postprandial period when healthy men consume lipid and dextrose meals of different size; 2) Both testosterone and cortisol experience a drop during the acute postprandial period, which is similar to what is expected based on the normal diurnal variation--feeding with lipid or dextrose meals does not appear to alter this pattern; 3) dextrose meals of either 75 g or 150 g result in a significant increase in serum insulin, in particular at 0.5 hr and 1 hr post-ingestion; 4) lipid meals have little impact on serum insulin.

## Background

Many investigators have sought to elucidate the hormonal response to feeding, as such an understanding may provide insight into important biological processes that occur in the postprandial state. Both the meal size [1,2] and macronutrient type [3-5] may impact the hormonal response. Although this ensuing hormonal response may be important to a variety of individuals (e.g., diabetics, those with metabolic syndrome, those attempting to lose body weight), active individuals engaged in regular exercise appear particularly interested in this area [6]. This may be due to the fact that the hormonal response to feeding may be related to anabolism, which may have a direct impact on exercise training-induced adaptations (e.g., muscle mass gain, glycogen resynthesis). With this in mind, many active individuals have adapted feeding strategies in attempt to favorably alter the circulating levels of these hormones. Specifically, some active individuals choose to consume high carbohydrate meals [7]; although, recommendations also include the consumption of high fat meals while restricting dietary carbohydrate [8,9].

Although much literature exists with regards to the postprandial hormonal milieu, data are conflicting with regards to the hormonal response following the ingestion of carbohydrate- and lipid-rich food [4,10-17]. Moreover, to our knowledge, no studies have compared the acute hormonal response to ingestion of carbohydrate and lipid meals of different size.

The hormones that appear to receive the most attention within the athletic world, in particular as related to feeding, are insulin, testosterone, and cortisol. Insulin has multiple physiological functions, ranging from the stimulation of blood glucose uptake into cells [18] to protein anabolism [19]. It is well documented that insulin significantly increases following ingestion of a carbohydrate rich meal [2,3,11,12,20], with more pronounced increases noted in those with impaired glucose tolerance [12]. Insulin has also been noted to increase following ingestion of a meal rich in saturated fat (~40 grams) [13], unsaturated fat (~26 grams) [12], and a ratio of saturated to unsaturated fat (52:59 grams) [17]. The above investigations included men with high fasting triglyceride levels (33 ± 7 years), a combination of healthy men and men with metabolic syndrome (age range: 20-33 and 18-49 years, respectively), and healthy men (27 ± 8 years), respectively. However, the insulin response to feeding has also been shown to be minimal when healthy men (age range: 20-25 years) ingest meals rich in saturated fats (~45 grams) [15]. Clearly, the population tested, as well as the type and quantity of macronutrient, may influence the postprandial insulin response with regards to both carbohydrate and lipid meals.

Related to testosterone, a well-described anabolic hormone involved in muscle tissue growth, a diet that is *chronically *high in fat appears to increase endogenous testosterone production [21]. However, acute intake of dietary fat results in a reduction in total testosterone [14,17]. Comparable findings are noted with consumption of acute carbohydrate meals, a finding documented in healthy men and male patients with chronic obstructive pulmonary disease [10], as well as in healthy and obese women [11]. Similar, although insignificant, reductions in total testosterone following a carbohydrate supplement have been reported in resistance-trained men [6].

The findings related to the catabolic hormone cortisol are somewhat similar to those for testosterone. That is, cortisol has been shown to significantly decrease following ingestion of a high fat meal in healthy men [4,17]. However, the literature is not in agreement with regards to the cortisol response to a high carbohydrate meal. Some investigations demonstrate significant increases in cortisol following high carbohydrate meals in healthy men [4], as well as in women with abdominal obesity [16]. This could potentially be due to the finding of increased insulin and subsequent decreased blood glucose--which in response may stimulate an increase in cortisol in an attempt to maintain glucose homeostasis [22]. Other studies note non-significant changes in cortisol with carbohydrate feeding in resistance-trained men [6], and in healthy women [16]. Such discrepancies may be a function of subject population [16], meal size, and carbohydrate type (e.g., complex versus simple) [23]. Moreover, a potential confound in this work is the fact that some studies involve an initial blood sample obtained in a fasted state [6,16], while others include a breakfast meal prior to obtaining the initial blood sample, which is then obtained close to mid-day when the actual test meal is administered [4,24]. Having a fundamental understanding of the circadian rhythm of both cortisol and testosterone [25,26], it appears important to obtain baseline blood samples in the morning while subjects are in a fasted state.

In the present investigation we compared the hormonal response to lipid and carbohydrate meals of different caloric content during the acute postprandial period. We hypothesized that the carbohydrate meals would result in the greatest increase in serum insulin, while the lipid meals would result in the greatest decrease in serum cortisol. These effects would be dependent on meal size (larger meals = greater response). We believed that the response for testosterone would be similar between meals--and would decrease during the postprandial period.

## Methods

### Subjects and Screening

Ten young, healthy men were initially recruited from the University of Memphis campus and Memphis community. One subject dropped from the study prior to completing all four meals testing days due to a loss of interest. The sample size was chosen based on prior work in this area of study using similar outcome variables, in particular with a cross-over design. All subjects were non-smokers, of normal body weight, normolipidemic (fasting triglycerides < 200 mg·dL^-1^), non-diabetic (fasting glucose < 126 mg·dL^-1^), with no history of diagnosed cardiovascular or metabolic disorders. Subject descriptive characteristics are presented in Table 1.

**Table 1:** Characteristics of 9 men.

Variable	Value
Age (yrs)	22 ± 2
Height (cm)	181 ± 8
Weight (kg)	82 ± 12
BMI (kg·m^-2^)	25 ± 4
Body fat (%)	19 ± 7
Waist (cm)	84 ± 9
Hip (cm)	103 ± 6
Resting heart rate (bpm)	68 ± 10
Resting SBP (mmHg)	117 ± 6
Resting DBP (mmHg)	66 ± 9

Data are mean ± SD.

During the initial visit to the lab, health history, medication and dietary supplement usage, and physical activity questionnaires were completed by subjects. The height, weight, and body composition of each subject was measured using a stadiometer, digital scale, and Lange skin fold calipers (via 7 site skinfold test and use of the Siri equation for estimating body density), respectively. Heart rate (via palpation) and blood pressure (via auscultation) were recorded following a 10 minute period of quiet rest. An explanation of dietary data recording was provided, along with data collection forms. Each subject was informed of all procedures, potential risks, and the benefits associated with the study. This was done through verbal and written form in accordance with the approved procedures of the University Institutional Review Board for Human Subjects Research and subjects provided written informed consent.

### Meal Testing

Subjects reported to the lab in the morning following a 10-hour overnight fast. The time of day for each subject was similar for all testing sessions in an attempt to control for diurnal variation in serum hormones. Upon arrival, subjects rested for 10 minutes and then a pre-meal blood sample was collected. On four different days, using a random order cross-over design, and separated by 3-7 days, subjects consumed one of four meals: dextrose at 75 grams (300 calories), dextrose at 150 grams (600 calories), lipid at 33 grams (300 calories), lipid at 66 grams (600 calories). The dextrose was delivered in powder form (NOW Foods, Bloomingdale, IL; 100% carbohydrate kcal; 100% sugar) mixed in water and the lipid consisted of heavy whipping cream (standard dairy grade; 100% fat kcal; 60% saturated fat, 30% monounsaturated fat, 10% polyunsaturated fat). We chose dextrose and whipping cream in an attempt to specifically include both pure carbohydrate and pure lipid. We have noted in our past studies that both drinks are fairly well tolerated by subjects; this was also the case in the present study. All drinks contained water, as follows: the 300 kcal drinks contained a total of 350 mL of fluid and the 600 kcal drinks contained a total of 700 mL of fluid. The amount of dextrose powder and whipping cream was weighed (laboratory grade balance) and measured prior to the mixing of each drink. The volume of water added to each drink (in order to bring the total volume to 350 mL or 700 mL) was measured in a graduated cylinder. All portions were mixed in a blender. Subjects were then provided 10 minutes to consume the assigned drink.

**Figure 1:**
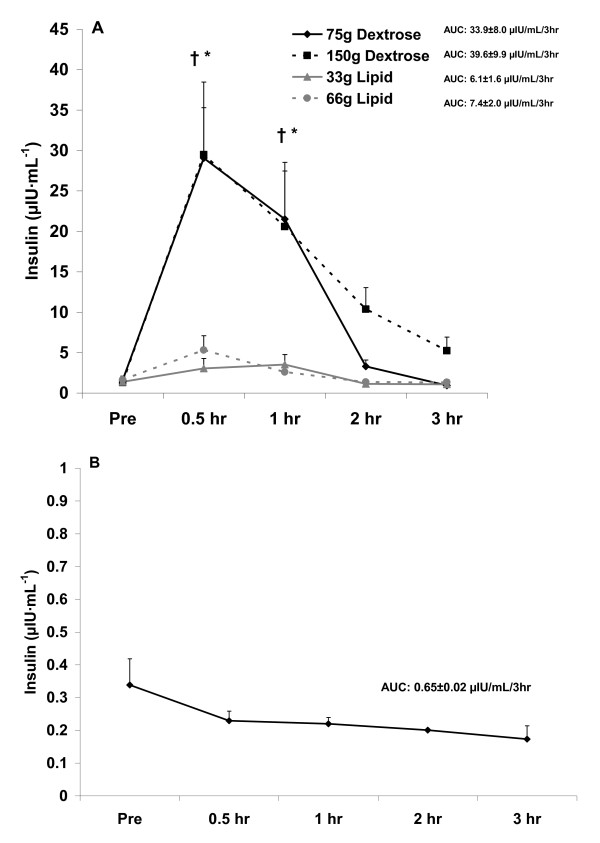
**Serum insulin before and after the consumption of a dextrose or lipid meal (A) and before and after a period of fasting (B)**. Data are mean ± SEM. †Meal × Time effect (p = 0.0003); higher at 0.5 hr and 1 hr compared to Pre for both dextrose meals; higher at 0.5 hr and 1 hr for both dextrose meals compared to both lipid meals (p < 0.05). Meal effect (p < 0.0001); both dextrose meals higher than both lipid meals (p < 0.05). *Time effect (p < 0.0001); higher at 0.5 hr and 1 hr compared to all other times (p < 0.05). AUC effect (p = 0.001); both dextrose meals higher than both lipid meals (p < 0.05).

**Figure 2:**
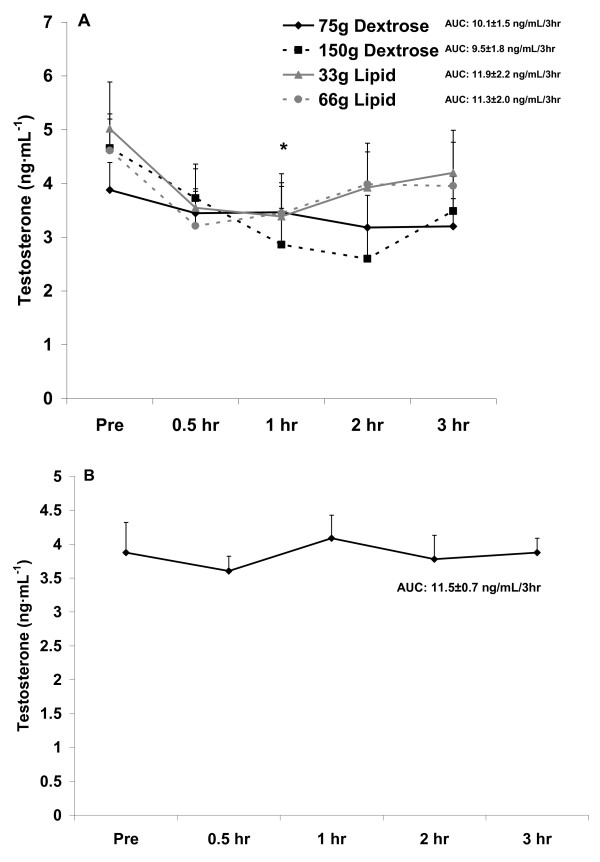
**Serum testosterone before and after the consumption of a dextrose or lipid meal (A) and before and after a period of fasting (B)**. Data are mean ± SEM. Meal × Time effect (p = 0.98). Meal effect (p = 0.39). *Time effect (p = 0.04); lower at 1 hr compared to Pre (p < 0.05). AUC effect (p = 0.85).

**Figure 3:**
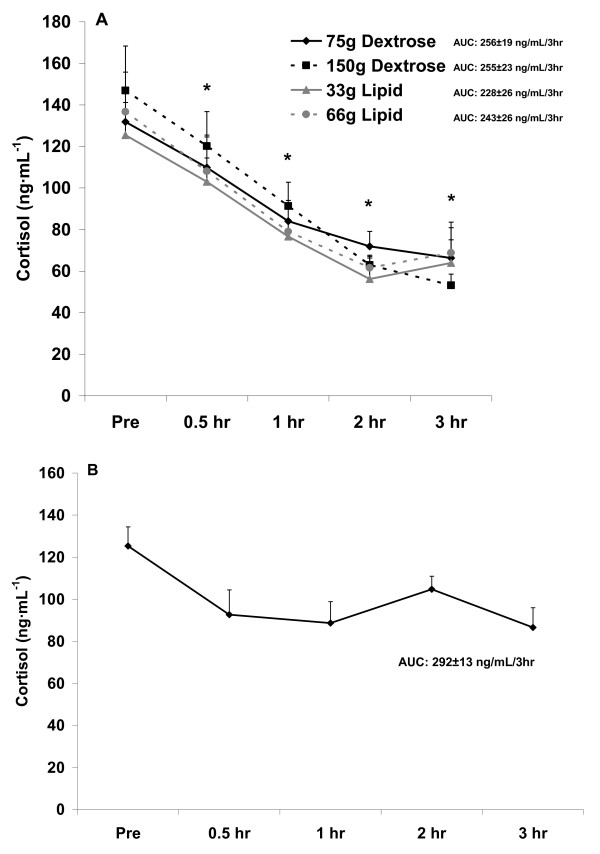
**Serum cortisol before and after the consumption of a dextrose or lipid meal (A) and before and after a period of fasting (B)**. Data are mean ± SEM. Meal × Time effect (p = 0.99). Meal effect (p = 0.65). *Time effect (p < 0.0001); lower at all times compared to Pre (p < 0.05). AUC effect (p = 0.84).

It should be noted that no placebo condition (no food) was provided in this investigation. This was partly due to the fact that the hormonal response to acute fasting is well described, with insulin remaining relatively stable over time [25], and both testosterone [26] and cortisol [25] falling during the morning hours. In addition, we have data from pilot work using a sample of 5 healthy men (mean age: 25 yrs), in which subjects reported to the lab in the morning hours in a 10 hour fasted state and remained fasted for a period of three hours so that blood could be collected and analyzed for insulin, testosterone, and cortisol. Our data from this pilot experiment corroborate the published findings. We have presented these pilot data in Figure 1B, 2B, and 3B, simply to use for visual comparison.

The postprandial observation period lasted three hours, during which time four additional blood samples were collected (0.5 hr, 1 hr, 2 hr, and 3 hr). Subjects remained in the lab or in close proximity during this period and expended very little energy (i.e., watched movies, worked on the computer, read). No other meals or calorie containing beverages were allowed during this period. Water was allowed *ad libitum *during the first test day and matched for all subsequent test days.

### Blood Collection and Biochemistry

Blood samples were obtained from subjects' forearm vein via needle and Vacutainer^®^. Following collection, blood samples were allowed to clot at room temperature for 30 minutes and then processed in a refrigerated centrifuge (2000 g for 15 min at 4°C) in order to obtain serum. Serum samples were stored at -70°C until analyzed for hormones of interest. Insulin, testosterone, and cortisol were all analyzed using enzyme linked immunosorbent assay (ELISA) techniques according to the manufacturer (Calbiotech, Spring Valley, CA).

### Dietary Records

Subjects were asked to maintain their normal diet and to record all food and beverage intake during the 24 hour period prior to each test day. Nutritional records were analyzed for total kilocalories, protein, carbohydrate, fat, vitamin C, vitamin E, and vitamin A (Food Processor SQL, version 9.9, ESHA Research, Salem, OR). Subjects were also asked to maintain their normal physical activity habits during the study period but to avoid strenuous exercise during the 24 hours preceding each test day.

### Statistical Analysis

For each hormone, the area under the curve (AUC) was calculated using the trapezoidal method as described by Pruessner et al. [27]. In addition, data were analyzed using a 4 (meal) × 5 (time) repeated measures analysis of variance (ANOVA). Significant interactions and main effects were further analyzed using Tukey's post hoc tests. Dietary variables were analyzed using a one-way ANOVA. All analyses were performed using JMP statistical software (version 4.0.3, SAS Institute, Cary, NC). Statistical significance was set at P ≤ 0.05. The data are presented as mean ± SEM, except for subject descriptive characteristics which are presented as mean ± SD.

## Results

Nine subjects successfully completed all meal testing. No statistically significant differences were noted for kilocalories (p = 0.34), grams of protein (p = 0.87), grams of carbohydrate (p = 0.50), grams of fat (p = 0.53), vitamin C (p = 0.76), vitamin E (p = 0.85), or vitamin A (p = 0.73). Dietary data are presented in Table 2.

**Table 2:** Dietary data of 9 men during the 24 hours before intake of a dextrose or lipid meal.

Variable	Dextrose 75 g	Dextrose 150 g	Lipid 33 g	Lipid 66 g
Kilocalories	2023 ± 237	2354 ± 242	1983 ± 206	1789 ± 181
Protein (g)	92 ± 11	102 ± 9	95 ± 13	88 ± 16
Carbohydrate (g)	261 ± 39	315 ± 41	248 ± 31	247 ± 33
Fat (g)	72 ± 11	81 ± 12	72 ± 13	57 ± 9
Vitamin C (mg)	64 ± 26	47 ± 11	40 ± 7	51 ± 13
Vitamin E (mg)	4 ± 2	4 ± 1	3 ± 1	3 ± 1
Vitamin A (RE)	267 ± 82	374 ± 110	228 ± 113	236 ± 102

Data are mean ± SEM.

No statistically significant differences noted for kilocalories (p = 0.34), protein (p = 0.87), carbohydrate (p = 0.50), fat (p = 0.53), vitamin C (p = 0.76), vitamin E (p = 0.85), or vitamin A (p = 0.73).

With regards to insulin, a meal × time effect (p = 0.0003) was noted, with values higher at 0.5 hr and 1 hr compared to Pre meal for both 75 g and 150 g dextrose meals, and higher at 0.5 hr and 1 hr for dextrose meals compared to lipid meals (p < 0.05). A meal effect was also noted for insulin (p < 0.0001), with both dextrose meals higher than lipid meals (p < 0.05). Finally, a time effect was noted for insulin (p < 0.0001), with values higher at 0.5 hr and 1 hr compared to all other times (p < 0.05). The AUC for insulin (p = 0.001) was higher for both dextrose meals compared to the lipid meals (p < 0.05). Insulin data are presented in Figure 1.

With regards to testosterone, no interaction (p = 0.98) or meal (p = 0.39) effect was noted. However, a time effect was noted (p = 0.04), with values decreasing during the postprandial period and being statistically lower at 1 hr compared to Pre meal (p < 0.05). No AUC effect was noted for testosterone (p = 0.85). Testosterone data are presented in Figure 2.

With regards to cortisol, no interaction (p = 0.99) or meal (p = 0.65) effect was noted. However, a time effect was noted (p < 0.0001), with values lower at all times during the postprandial period as compared to Pre meal (p < 0.05). No AUC effect was noted for cortisol (p = 0.84). Cortisol data are presented in Figure 3.

Although we did not include a "no food" placebo condition in the present design, we have conducted a pilot experiment in which blood was collected from 5 healthy men at the same times as in the present study, while men remained fasting, and analyzed for the hormones of interest. When comparing findings from the present study to those of the pilot experiment, the following are noted: Insulin values were relatively unchanged in response to the no food condition (Figure 1B) and although no increase of statistical significance was noted with the lipid meals, values for insulin did increase slightly, in a dose dependent manner (Figure 1A).

The noted decrease in testosterone (Figure 2A), which was not different between meals, is not observed in the fasted state (Figure 2B). However, for cortisol the decrease is more pronounced with feeding (Figure 3A), as values are relatively stable between 0.5 hr and 3 hr when fasting (Figure 3B). This may be related to the rise in cortisol during a fasting period in an attempt to maintain blood glucose [25]. Collectively, it appears that feeding with either lipid or carbohydrate is associated with a decrease in circulating testosterone and cortisol, without differences noted between meals.

## Discussion

Findings from the present study indicate that 1) little difference is noted in serum testosterone or cortisol during the acute postprandial period when healthy men consume lipid and dextrose meals of different size; 2) Both testosterone and cortisol experience a drop during the acute postprandial period (regardless of the meal consumed; regardless of the insulin response), which is similar to what is observed during an acute fasting state and follows the normal diurnal variation of these hormones; 3) dextrose meals of either 75 g or 150 g result in a significant increase in serum insulin, in particular at 0.5 hr and 1 hr post-ingestion; 4) lipid meals have little impact on serum insulin during the acute postprandial period.

Considered collectively, ingestion of either carbohydrate (in the form of dextrose) or lipid (in the form of heavy whipping cream) does not differently impact the hormonal response to feeding, as measured by serum testosterone and cortisol. However, serum insulin is largely impacted by dextrose feeding, as was expected based on the acute rise in serum glucose that occurs with such feeding [28]. While the increase in circulating insulin may be viewed as welcome for some individuals (e.g., active individuals attempting to resynthesize muscle glycogen [29] or favoring the anabolic activity of insulin [30]), chronic ingestion of high quantities of simple sugar may not be optimal for overall health, as it may lead to weight gain [31], impaired insulin sensitivity [32], and other untoward effects [33] in certain individuals.

Cortisol decreased to a similar extent following carbohydrate and lipid meals, despite a drastically different insulin response. While some authors have reported no change in cortisol following a high carbohydrate meal in active and sedentary men [2,6,16], others have noted significant increases in cortisol, in particular when compared to meals rich in fat [4,16]. Martens et al. noted that when healthy men consume a carbohydrate meal consisting of 18% of daily energy requirements, a significant increase in cortisol is observed when compared to a fat and protein meal of similar hedonic values [4]. It has been postulated that this relative increase in cortisol following carbohydrate feeding occurs due to the ensuing stress resulting from a spike in blood glucose, and the subsequent rise in serotonin, which then leads to an increase in cortisol [4].

Our findings, as well as those of others [6,16], do not support an increase in cortisol in healthy men and women consuming a high carbohydrate meal--possibly due to more tightly regulated blood glucose control in a population of healthy individuals. However, Vicennati and colleagues demonstrated an increase in cortisol when women with abdominal obesity consumed a high (89%) carbohydrate meal, as well as after consumption of a mixed protein/lipid meal (43% protein and 53% lipid) in women with peripheral obesity [16]. While we noted no differences in postprandial cortisol response regardless of meal type or size, our subjects were young and healthy men and consumed only an isolated morning meal. As with many aspects of human nutrition, differences in subject population may impact findings.

To our knowledge, no other studies have investigated the effects of different macronutrients, provided at different caloric values, on insulin, testosterone, and cortisol. Aside from insulin, which increases significantly in response to carbohydrate but not lipid ingestion, no differences were noted in testosterone or cortisol in response to macronutrient ingestion of different type or meal size. Specifically, both testosterone and cortisol decreased in a pattern that follows the normal diurnal variation in these hormones. As discussed above, our results for cortisol agree with some prior reports, while our findings for decreased testosterone following meals rich in carbohydrate [2,10,11] and fat [14,17] are also supported. A finding of interest in the present study is the fact that the response for these hormones does not differ based on caloric content of the meal.

Although we did not make a direct comparison between our findings with the four meals and those involving a fasting condition, the drop in testosterone (Figure 2) and cortisol (Figure 3) with feeding appears more pronounced than with fasting. For testosterone, this may be viewed as negative, as increased levels of testosterone would be favored during the postprandial period to allow for anabolism [34]. For cortisol, a further lowering during the postprandial period may be viewed as positive, as lower cortisol may be associated with decreased proteolysis [35]--also important when considering anabolism. However, despite these findings, no differences existed for meal type or size with regards to testosterone or cortisol. With regards to cortisol and the further reduction of this hormone following meal consumption as compared to when in a fasted state, a calorie load of some unknown and relatively small value may be adequate to minimize the rise in this hormone--which may be in direct response to a drop in blood glucose and an attempt for cortisol to assist in maintaining glycemia while in a fasted state [22].

Admittedly, we do not fully understand what such acute changes in hormone concentrations mean as related to overall health and muscle tissue growth. Clearly, testosterone has been reported to increase following exercise [36], and is believed to be a major contributor to muscle mass gain [37]. It is logical to assume that elevated testosterone may equate to a greater degree of muscle growth over time; hence, methods of increasing testosterone via food intake appear appropriate. However, when exercise is followed by the consumption of carbohydrate and/or protein, testosterone values fall below resting levels in resistance-trained men [38,39]. This drop in testosterone is not observed in trained men who consume a placebo following exercise [6,39]. Despite the potential drop in testosterone during the acute postprandial period, carbohydrate/protein supplementation occurring two hours before exercise and immediately post-exercise, results in a peak of serum insulin concentrations by 500% above resting values within 45 minutes of ingestion [39]. Considering the multiple components and systems involved in regulating both anabolic and catabolic processes, the acute changes in circulating hormones from macronutrient consumption must be viewed with caution. That is, although testosterone may be acutely decreased with feeding, avoiding the ingestion of nutritious foods (in particular, post-exercise) may prove counterproductive with regards to influencing other anabolic hormones (e.g., insulin), as well as other aspects of human health and recovery (e.g., cellular immunity, glycogen resynthesis).

It is important to note some limitations of this work. First, we used a sample of healthy men, with measurements obtained in a fasted state. It is possible that subjects with known disease, and/or women, may have responded differently. Second, testing was conducted in the morning hours, in an attempt to control for the diurnal variations in hormones, and measurements ceased three hours following meal ingestion. Different results may have been obtained if testing was conducted at a different time of day [4] and/or if measurements extended beyond the three hour post meal time [1,12,13]. Third, our study only involved the ingestion of isolated carbohydrate (in the form of dextrose) and lipid (in the form of heavy whipping cream) meals. The inclusion of protein meals [40], or mixed meals [1], may have resulted in different findings. Fourth, we only included a measure of total testosterone, and not free testosterone, which is the most biologically active state of testosterone comprising about 0.2-2% of total testosterone [34]. It is possible that free testosterone may have responded differently to feeding. Fifth, other hormones involved in anabolism and catabolism, such as growth hormone, were not measured. Measurement of additional hormones may have provided further insight into the impact of feeding on postprandial hormonal response. Finally, the inclusion of exercise within the research design could have introduced another variable which may have impacted our findings [6]. Further research in this area may consider the above limitations in order to improve upon the study design.

## Conclusions

Our data indicate that acute feeding of either lipid or carbohydrate of varying size has little impact on serum testosterone or cortisol during the acute postprandial period. Serum insulin is significantly increased by carbohydrate feedings, but not lipid feedings. Future work should consider the inclusion of older and metabolically compromised individuals, as well as women, in an effort to determine their response to single macronutrient feeding of different loads. These studies may also consider the use of multiple meals of a particular macronutrient to gather data regarding how these hormones are affected during a 24 hour cycle. This would further clarify whether the changes in cortisol and testosterone are indeed impacted by feeding or if they simply follow their diurnal cycle.

## Competing interests

Financial support for this work was provided by the University of Memphis. The authors declare no competing interests.

## Authors' contributions

RJA was responsible for literature review and manuscript preparation. RJB was responsible for the study design, biochemical work, statistical analyses, and manuscript preparation. Both authors read and approved of the final manuscript.
